# AmpliconTyper – a tool for analysing ONT multiplex PCR data from environmental and other complex samples

**DOI:** 10.1099/mgen.0.001421

**Published:** 2025-09-10

**Authors:** Anton Spadar, Jaspreet Mahindroo, Catherine Troman, Michael Owusu, Yaw Adu-Sarkodie, Ellis Owusu-Dabo, Dilip Abraham, Blossom Benny, Karthikeyan Govindan, Venkata Raghava Mohan, Zoe A. Dyson, Nicholas Grassly, Kathryn E. Holt

**Affiliations:** 1 Department of Infection Biology, Faculty of Infectious and Tropical Diseases, London School of Hygiene & Tropical Medicine, London, UK; 2 MRC Centre for Global Infectious Disease Analysis, Department of Infectious Disease Epidemiology, School of Public Health, Imperial College London, London, UK; 3 College of Health Sciences, Kwame Nkrumah University of Science and Technology, Kumasi, Ghana; 4 The Wellcome Trust Research Laboratory, Christian Medical College, Vellore, India; 5 Department of Community Health And Development, Christian Medical College, Vellore, India; 6 Wellcome Sanger Institute, Wellcome Genome Campus, Hinxton, UK; 7 Department of Infectious Diseases, School of Translational Medicine, Monash University, Melbourne, Australia

**Keywords:** amplicons, environmental surveillance, multiplex PCR, Oxford Nanopore Technologies

## Abstract

Amplicon sequencing is a popular method for understanding the diversity of bacterial communities in samples containing multiple organisms as exemplified by 16S rRNA sequencing. Another application of amplicon sequencing includes multiplexing both primer sets and samples, allowing sequencing of multiple targets in multiple samples in the same sequencing run. Multiple tools exist to process the amplicon sequencing data produced via the short-read Illumina platform, but there are fewer options for long-read Oxford Nanopore Technologies (ONT) sequencing, or for processing data from environmental surveillance or other sources with many different organisms. We have developed AmpliconTyper (v0.1.28, DOI: 10.5281/zenodo.15045111) for analysing multiplex amplicon sequencing data from environmental (e.g. wastewater) or similarly complex samples, generated using ONT devices. The tool uses machine learning to classify sequencing reads into target and non-target organisms with very high specificity and sensitivity. The user can train models using public and/or user-generated data, which can subsequently be applied to analyse new data. The tool can also generate amplicon consensus sequences, as well as identify SNPs and report their genotype implications, such as association with lineages or antimicrobial resistance (AMR). The tool is freely available via Bioconda and GitHub (https://github.com/AntonS-bio/AmpliconTyper). AmpliconTyper allows robust identification of target organism reads in ONT-sequenced environmental samples and can identify user-specified lineage or AMR markers.

**Impact Statement**

AmpliconTyper (v0.1.28) is a tool that enables users to analyse amplicon sequences generated by targeted amplification followed by Oxford Nanopore Technologies sequencing, for environmental or other similarly complex samples. The analysis includes mapping of reads to target amplicon sequences, classification of each sequenced read as either originating from a target or non-target organism, followed by identification of user-specified SNPs and generation of an interactive report summarizing the findings. The strength of AmpliconTyper lies in its ability to train a machine learning model using public data to create sequencing read classification models tailored to a user’s application. AmpliconTyper is designed specifically to work with extremely noisy data that includes a large share of off-target amplification reads such as those encountered in environmental surveillance applications.

## Data Summary

For the purpose of designing and testing AmpliconTyper, we have used two datasets. The first consisted of 69 *Salmonella enterica* serovar Typhi (*S*. Typhi) and 10,303 other *Enterobacteriaceae* whole-genome sequencing Oxford Nanopore Technologies nanopore sequencing libraries (Data S1, available with the online Supplementary Material) from the National Center for Biotechnology Information (NCBI) Sequence Read Archive [1]. We used these data to evaluate the performance of different classifier models and to train a model for our use case, i.e. amplicon-based detection of *S*. Typhi from environmental surveillance samples [2].

In addition, to further evaluate the performance of AmpliconTyper in our use case, we have applied an amplicon sequencing protocol [2] to generate amplicon data for *S*. Typhi using two *S*. Typhi isolates (NCBI accessions SRR5949979 and SRR7165748] provided by Satheesh Nair (UKHSA) [3]. We also used the same protocol to the pooled sample of the American Type Culture Collection (ATCC) consisting of *S*. Paratyphi A (ATCC 9150D), *S*. Paratyphi B (ATCC-BAA-1250D), *S*. Paratyphi C (ATCC-BAA-1715D), *Aeromonas hydrophila* (ATCC-7965D), *Klebsiella pneumoniae* (ATCC-BAA-1706D) and *Citrobacter freundii* (ATCC-8090D) chosen for their close relationship to *S*. Typhi. The test data for classification are available at https://github.com/AntonS-bio/AmpliconTyper/tree/main/test_data. Newly generated data were deposited in the European Nucleotide Archive project PRJEB81565.

Supplementary data and files are available in the Zenodo repository (DOI: 10.5281/zenodo.15045111) [4].

## Introduction

PCR is a well-established method for amplifying the segment of DNA lying between two primer sequences. The resulting amplicon can subsequently be subjected to sequencing to determine its precise DNA sequence, providing information on both the presence of, and variation within, the targeted segment. Multiplex PCR extends this approach by using multiple PCR primer pairs in the same reaction to generate multiple target amplicons. By multiplexing both primer sets and samples, the lab costs are further reduced with the additional benefit of primer multiplexing giving improved sensitivity and specificity of organism detection due to simultaneous detection of multiple genetic regions from a single target organism [5,6].

We have developed the AmpliconTyper tool (v 0.1.28, available at https://github.com/AntonS-bio/AmpliconTyper), to detect a specific organism based on amplicon sequences from environmental samples generated using Oxford Nanopore Technologies (ONT) devices and also to identify lineage-defining and antimicrobial resistance (AMR)-linked SNPs [7,8]. Our use case for development is detection and typing of *S*. Typhi, which extends previous *S*. Typhi qPCR-based surveillance work [5,9] by integrating ONT sequencing of amplicons to more precisely detect the pathogen, and by expanding the number of *S*. Typhi-specific amplicons from 1 to 16 targeting 22 SNPs associated with 17 lineages and 4 AMR-associated determinants [10]. While benefiting from long reads, ONT sequencing currently suffers from substantially higher base-calling error rates compared to Illumina devices (e.g. 0.7% error rate in amplicons sequenced using R10.4.1 flowcell compared with 0.1% for Illumina) [11]. There are multiple tools for analysis of common amplicon data such as bacterial 16S rRNA sequencing [12,15] and 16S-ITS-23S [16], as well as metagenomic data [13] and amplicons [17]. However, there are fewer tools [18] for the classification of specific ONT sequencing amplicons against established genotypes, i.e. below species or serovar level. The main difference of AmpliconTyper is that it focuses on single organisms and its predefined genotypes instead of overall diversity within sample or clustering of sample based on genetic distances. AmpliconTyper also reports the results focusing on the target organism in a format that we tailored for environmental surveillance based on user experience. AmpliconTyper is a flexible tool that can be used to identify ONT reads from a specific target organism in environmental or other samples with multiple diverse organisms and to detect epidemiologically and phenotypically important alleles.

## Theory and implementation

### Overview

**Fig. 1.**
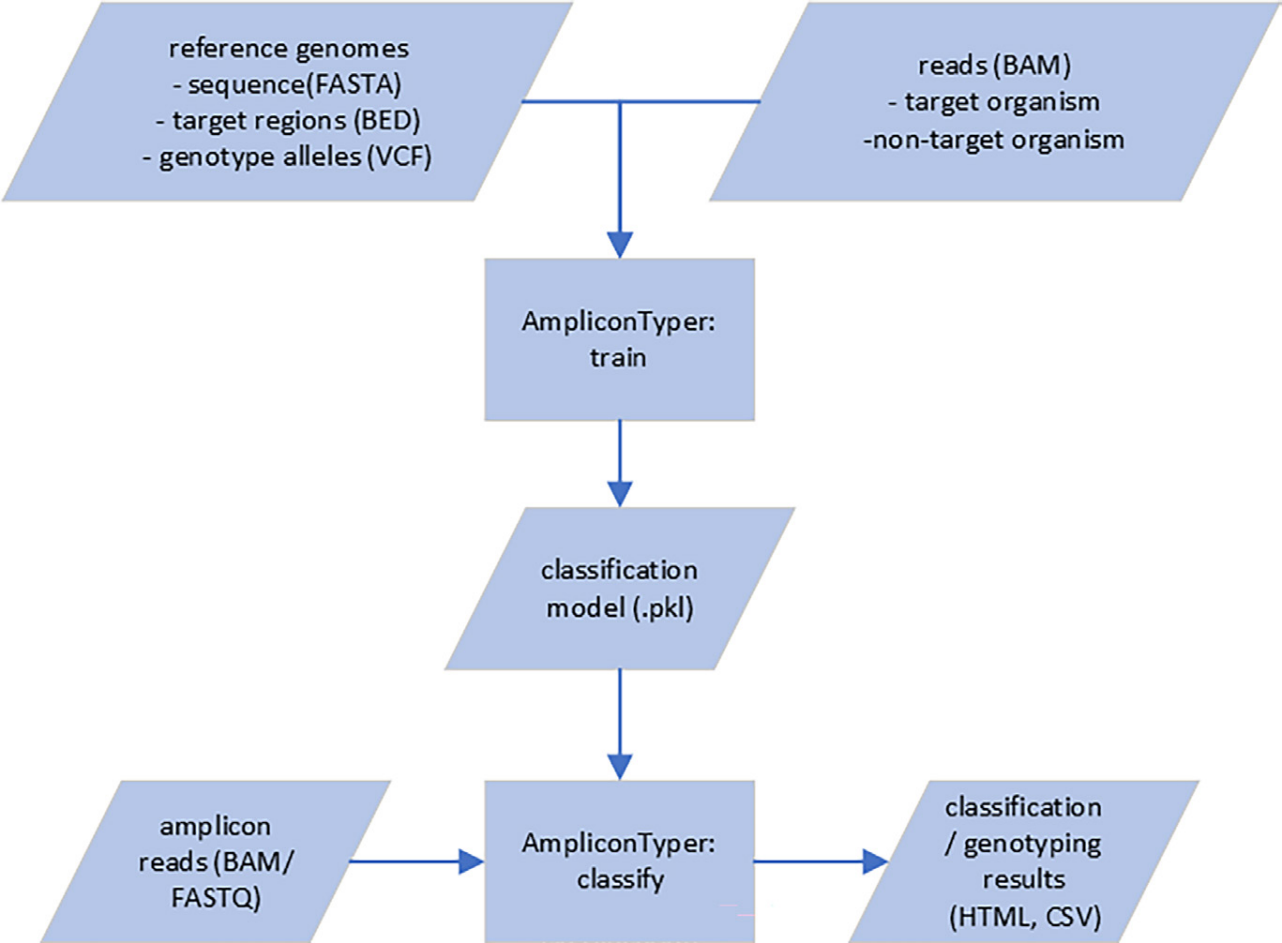
General workflow of the training and classification.

**Fig. 2.**
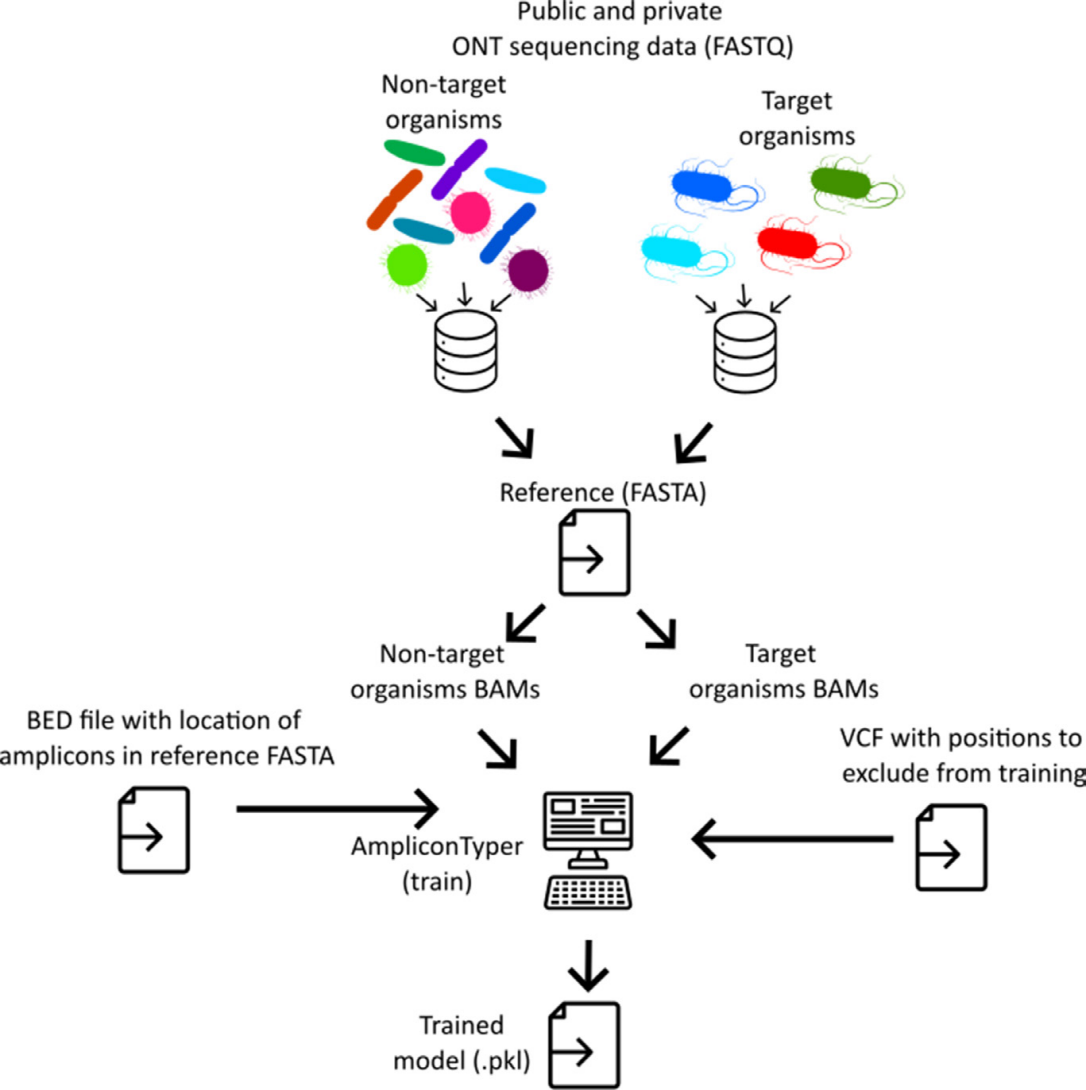
Overview of the training process. FASTQ files of whole-genome sequencing (WGS) data from public and/or user databases are aligned to the desired amplicon sequences. The resulting BAM files, representing target and non-target organism sequences, are fed to the AmpliconTyper ‘train’ function, which trains an ML classifier and outputs it to a model file (.pkl) that can be used to classify new data (see **Fig. 2**).

**Fig. 3.**
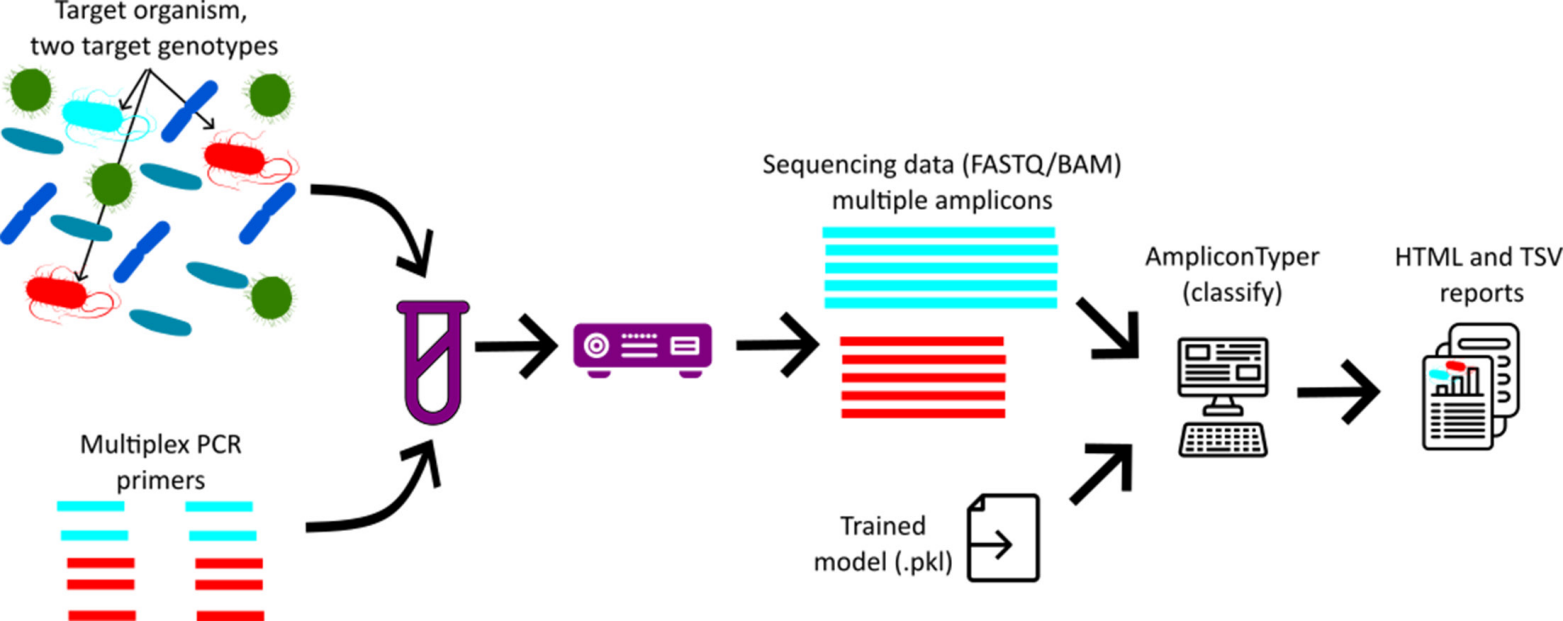
Genotyping workflow. The sample DNA is amplified in a single multiplex PCR. The resulting amplicons are sequenced on an ONT device, and the sequence reads are analysed using AmpliconTyper. The output is a report (File S1) describing amplification results for each primer and summary of mutations in the PCR products.

We define the target organism as an organism that the user is trying to detect in a given sample. We define non-target organisms as all other organisms that may be present in the same sample. AmpliconTyper is designed to differentiate between ONT-sequenced amplification reads originating from target and non-target organisms. The AmpliconTyper has two main functions: train and classify (Fig. 1). The former trains a machine learning (ML) model to distinguish ONT sequencing reads from target vs non-target organisms (Fig. 2). The latter classifies every read in an input file [FASTQ or BAM (binary alignment map) format] [19,20] as target or non-target organism based on this model and generates a report summarizing the results (Fig. 3). Users can also supply AmpliconTyper with a variant call format (VCF) file [21] containing a list of genotypes and AMR-associated SNPs which, if identified in the data, are listed in the report [21]. Additionally, if amplicons targeting accessory genes are included in the sequencing assay (e.g. acquired AMR genes), AmpliconTyper can call these as present/absent or with allelic variation, to support subtyping of the detected target pathogen. The version described here is v0.1.28 (DOI: 10.5281/zenodo.15045111).

### Training

AmpliconTyper relies on a trained ML model to classify ONT amplicon sequencing data from environmental and similarly complex samples. The training process is summarized in Figs 2 and S1. To train the model, the user needs to provide the corresponding function ‘*train*’ with:

- The reference sequences are provided as a FASTA file, and this can be either the whole reference genome of target organism or just the amplicon sequences from the reference genome of target organism.
- BED file specifying which regions in the reference sequence (a) are expected to be amplified [22].
- Directory with alignment of ONT reads to (a) from non-target organisms in BAM format [19]. This material can be both amplicons and WGS. The alignments should be generated using ‘*minimap2 -ax map-ont reference.fasta input.fastq*’.
- Directory with alignment of ONT reads to (a) from pure genomic material of target organism in BAM format (these can be from single or multiple genotypes). This material can be both amplicons and WGS. The alignments should be generated using ‘*minimap2 -ax map-ont reference.fasta input.fastq*’.
- VCF file [21] with list of positions in reference sequences (a) that should be excluded from training, as well as any genotype- or AMR-associated positions [21].

We have opted to use BAM files for training rather than FASTQs based on our experience. The bottleneck in the training of our model for *S.* Typhi was the downloading of FASTQs from the European Nucleotide Archive which took over a week using Aspera Connect (v 4.2.8.540) [23,24]. Another constraint was the computer storage space; we could not store all FASTQs at the same time. For this reason, we downloaded each sample’s FASTQs, mapped them to amplicon sequences and deleted the FASTQ before proceeding to the next sample. This generated fairly small BAM files resolving the storage constraint. While downloading BAMs, we experienced multiple download errors and had to redownload the failed samples. While the download and mapping process can be included in AmpliconTyper, we think it will be difficult to do so given the diversity of ways in which users may prefer to download the data.

Since AmpliconTyper is designed for applications such as environmental surveillance where samples contain a mixture of organisms, samples used to generate BAM files should reflect the likely diversity of organisms in the sample sources. For this reason, we recommend that users supply AmpliconTyper not only with user-generated amplicon data, but also with publicly available WGS ONT read data mapped to target sequences (Fig. 2). As WGS data will contain reads that only partially overlap the target amplicon (Fig. S2), it is essential to discard such reads from training; otherwise, the model simply learns that reads with large numbers of missing nucleotides are from non-target organisms. When applying the trained model to new data, we also discard reads with one or more missing nucleotides either at the start or end of the reference sequence, though this can be overridden. As a consequence of using only reads that fully span amplicons, the public WGS data will typically have many fewer usable reads than the mean depth of coverage (Fig. S2). For this reason, we recommend using as many public sequencing libraries as possible. For our exemplar work on *S*. Typhi, we used 10,301 non-Typhi and 68 *S*. Typhi WGS ONT libraries available via National Center for Biotechnology Information’s Sequence Read Archive database supplemented with one *S*. Typhi and two *S*. Paratyphi libraries that we generated (Data S1) [1].

The training uses a logistic regression model with stochastic gradient descent, implemented as SGDClassifier with log-loss function in scikit-learn v1.5.2 [25,26]. We decided to use an ML classifier for read classification because it simplifies the creation of a classifier model. An alternative approach can be to call SNPs from aligned reads and classify reads either based on specific SNPs or nucleotide distance between individual reads and reference. The two approaches are very similar in practice, but training ML classifiers on mapped reads does not require the user to explicitly define SNPs or nucleotide distance thresholds. Furthermore, the ML classifier also automatically adjusts the model parameters to account for ONT sequencing errors. The downside of this approach is the need to process large volumes of raw read data, and that if the sequencing error rate is substantially different from the training dataset, the results can be incorrect.

**Fig. 4.**
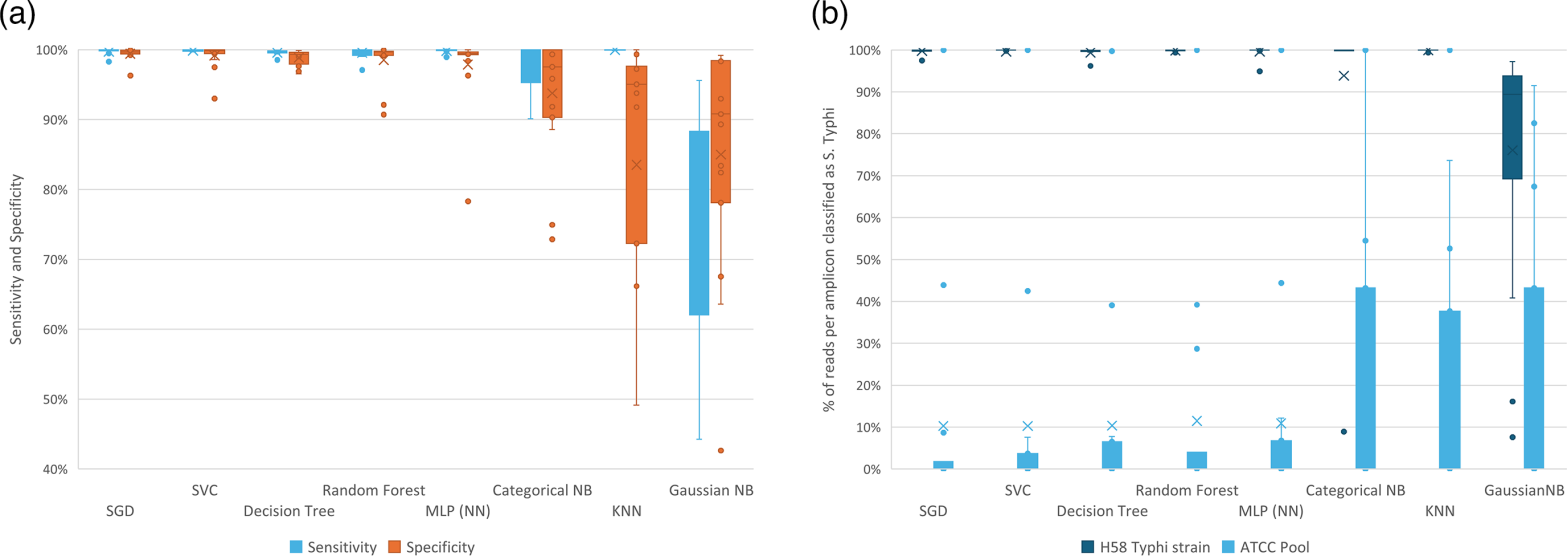
Comparison of classification models. (**a**) The training dataset consisted of 78 target organism samples and 10,303 non-target organism samples (mix of *S.* Typhi and non-*S*. Typhi ONT reads). (**b**) Each trained classifier from (**a**) was tested against newly generated data consisting of one sample of *S*. Typhi strain H58 and one sample of pooled *S*. Paratyphi A, B and C; *A. hydrophila*; *K. pneumoniae*; and *C. freundii* from ATCC. Of the 63,903 sequencing reads generated from the AATC pool, 58,832 mapped to the target amplicons, and of the 159,708 *S*. Typhi H58 sequencing reads, 155,749 did.

To train the model, the classifier first converts BAM files into a binary matrix with five columns per each nucleotide in the amplicon reference sequence to accommodate all possible nucleotide calls (A, C, T, G) and each row representing a single read from BAM files [27,28]. We generate one BAM matrix per amplicon and train a model for each amplicon independently of others, but trained models are stored in one file. This classifier achieved much better performance compared to other tested classifiers including ensemble approaches (Fig. 4). The outlier amplicon in Fig. 4(b) is the off-target amplification of a single gene in *S*. Paratyphi C, which was poorly represented in the training set. The training process consists of splitting the data into a training set (80% or 5,000 of reads, whichever is lower) and a test set (20% or 5,000 of reads, whichever is lower) with sensitivity and specificity as the model evaluation parameters. Importantly, all positions specified in the input VCF file (Fig. S1) are ignored during both training and classification [21]. This is because some positions in the amplicons include multiple alleles that define lineages or AMR-linked mutations, and their inclusion reduces reliability of the classification.

The above approach cannot deal with instances where the target organism sequence has no full-length homologues in non-target organisms (defined as fewer than five reads), for example, due to recombination events. In field samples, we cannot assume that all reads mapping to such amplicons come from target organisms because some may come from uncultured bacteria. To accommodate such cases, AmpliconTyper uses the GaussianMixture model from the scikit-learn package to determine the distribution of nucleotide distances (Hamming distance) between the training target organism reads and the reference sequence (excluding known variable sites from the VCF). If sequencing was perfect, all sequencing reads would be identical to the DNA sequence of the isolate used, but because of sequencing errors, each read will be slightly different. The GaussianMixture captures the distribution of these differences. AmpliconTyper uses this distribution to classify new reads based on whether their distance from reference is within 10% of closest reads used in training. This approach delivers low specificity and sensitivity which are not *a priori* quantifiable. To mitigate this, when classifying new data, AmpliconTyper reports the nucleotide differences between reads classified as the target organism and the reference sequence.

Once the model is trained, it is saved into a Python object serialization file (pickle file) that serves as input for the ‘*classify*’ function. The pickle file [29] is a binary file that users cannot explore or modify other than through functions ‘*classify*’, ‘*train*’ and ‘*genotyper_utilities*’ provided with AmpliconTyper.

Function ‘*genotyper_utilities*’ can be used to extract information from the trained model file and is under active development. It currently provides functionality to extract SNPs and amplicon reference sequences from the model files.

### Classification of new data

The ‘*classify*’ function applies the trained model to new data (Figs 3 and S3). It requires the following:

- Model pickle file (generated during model training as described above),
- Either a FASTQ or BAM file with reads to classify [19,20].

The classifier first maps the sequencing data to the reference sequence using minimap2 v2.1 [30] and then applies pre-trained models described above to each read in order to assign reads to one of the two categories: target organism or non-target organism. For each amplicon, the reads classified as the target organism are used to generate a consensus sequence. The consensus sequence is generated by taking the most common value (nucleotide or deletion) across all target organism reads for each position in the amplicon. Each position in consensus is compared to the corresponding position in the reference sequence, and the differences are reported to the user. Importantly, sequencing errors, especially in homopolymer regions, can lead to problems in generating accurate consensus. When interpreting the results, users should pay special attention to coverage.

We have included an option to assign genotype or AMR labels to specific alleles to facilitate the interpretation of AmpliconTyper outputs for amplicon-based genotyping. The allele information is supplied during model training and is stored within the model. We have opted to use the ‘ID’ field of the VCF file to store this information (Data S2) [21]. The value of the ID field is assumed to consist of two parts: allele association and allele type. For example, ‘4:GT’ means that the alternative (ALT) allele at this site implies genotype (GT) 4, whereas ‘gyrA_D87G:AMR’ means that the ALT allele implies AMR genotype gyrA_D87G. Positions with neither the ‘:GT’ nor ‘:AMR’ suffix are treated as not informative in the AmpliconTyper genotype report.

The results of classification are written to an HTML-formatted report (File S1) which provides the summary of results, the genotypes supported by the data, a detailed per-sample report of mapping results and identified differences from reference sequence/s.

The training and classification functionality can be tested using the provided test data (DOI: 10.5281/zenodo.15045111). Once AmpliconTyper has been installed and the test data extracted from archive, the training function can be tested using *train -t amplicons.bed -p ./positive_bams/ -n ./negative_bams/ -v amplicons.vcf -o ./output/ --cpus 1 r amplicons.fna*, and the classification function can be run using *classify -f ./test_data/fastqs/ -b ./test_data/bams/ -d ./test_data/metadata.tsv -c Description -o test_report.html -m ./test_data/typhi_v8.pkl*.

### Performance

We used AmpliconTyper on a Linux server [64 GB RAM, 8 Intel Xeon E5-2640 central processing units (CPUs)], but model training can, in principle, be done on a mid-level desktop, and classification is designed to work on a mid-level desktop computer (Table 1). To benchmark the AmpliconTyper training function, we used a reduced *S*. Typhi dataset which consists of 2,000 individual BAM files with negative training data and 78 BAM files with positive data. To benchmark alternative algorithms for the classification step (Fig. 4), we used 8 samples of multiplex amplicon sequencing of *S*. Typhi genomic DNA with 904,795 mapped reads (241 MB).

**Table 1.** Performance of training and classification on different computers

	Server (8 CPUs)	Desktop (1 CPU)
**CPU type**	Intel Xeon E5-2640	Intel i3-7100
**Operating system**	Linux	Linux ran via Windows WSL
**Training data loading**	~10 min	~1.2 h
**Model training**	~2 min	~2 min
**Memory peak**	~2.5 GB	~0.5 GB
**Classification of 230 MB of new data**	~2.5 min	~2 min

## Discussion

AmpliconTyper offers a simple way to detect epidemiologically relevant markers for a target organism from ONT amplicon sequencing of environmental or similarly contaminated samples. The AmpliconTyper is organism agnostic, relies on standard input file formats and can enrich output with identification of specific genotype and AMR alleles. A separate tool, EnviroAmpDesigner (https://github.com/AntonS-bio/EnviroAmpDesigner), was also developed to design the primers used in this study. EnviroAmpDesigner is meant for designing multiplex PCR primer panels for genotype identification from environmental surveillance and other complex sources. The two tools work independently, and AmpliconTyper can work with primers designed using other tools.

AmpliconTyper uses standard input files such as FASTA, VCF, BED and BAM that should be familiar to potential users [19,21, 22]. The output, while simple in appearance, is informative and gives the user both a quick summary and detailed results.

For installation of the AmpliconTyper, we strongly encourage using a package manager (conda or mamba) to instal the tool from Bioconda, but AmpliconTyper can also be installed directly from its GitHub repository (https://github.com/AntonS-bio/AmpliconTyper). Once installed, the tool exposes two commands - ‘*train*’ and ‘*classify*’ -both of which are meant to be run via command line. The tool can be used with either Linux or MacOS, and Windows 10 or higher (via the Windows Subsystem for Linux).

The main limitation of the AmpliconTyper is that it classifies the reads into two categories: target and non-target. While we plan to introduce support for multiple classification classes in the future, currently, multiple organisms must be modelled separately.

Another limitation stems from the requirement for reliable training data, with sufficient high-quality data confirmed as target and non-target. When public data is relied on, care must be taken to assess the quality of the sequence data and of the target/non-target labels. To assist users in identifying issues with the taxonomic labelling of training data, the training AmpliconTyper function outputs a list of samples with misclassified reads and the number of reads from each that were misclassified.

## Conclusions

AmpliconTyper offers a dedicated tool for analysis of multiplex ONT amplicon sequencing data from environmental or other samples with multiple organisms present. It relies on standard genomic data formats, provides a comprehensive results report, supports inference of genotype and AMR from alleles in the data and permits model training using public or user-generated data. AmpliconTyper fills an important gap in the bioinformatics toolkit, and we look forward to continuing its improvement with community feedback.

## Availability and requirements

Project name: AmpliconTyper

Project home page: https://github.com/AntonS-bio/AmpliconTyper.git

Operating system(s): Linux, MacOS or Windows 10+ with Windows Subsystem for Linux

Programming language: Python v3.10.12

Other requirements: None

Licence: GNU GPL v3

## Supplementary material

10.1099/mgen.0.001421 Uncited Supplementary Material 1.

## Abbreviations

AMR: antimicrobial resistance
ATCC: American Type Culture Collection
BAM: binary alignment map
CPU: central processing unit
ML: machine learning
NCBI: National Center for Biotechnology Information
ONT: Oxford Nanopore Technologies
*S.* Typhi: *Salmonella enterica* serovar Typhi
VCF: variant call format
WGS: whole-genome sequencing
