# Supplementary material for: AmpliconTyper – a tool for analysing ONT multiplex PCR data from environmental and other complex samples
Source: Microb Genom. 2025 Sep 10;11(9):001421. doi: 10.1099/mgen.0.001421 (PMC12452170; doi:10.1099/mgen.0.001421)
Supplement: Uncited Supplementary Material 1. [file mgen-11-01421-s001.pdf]

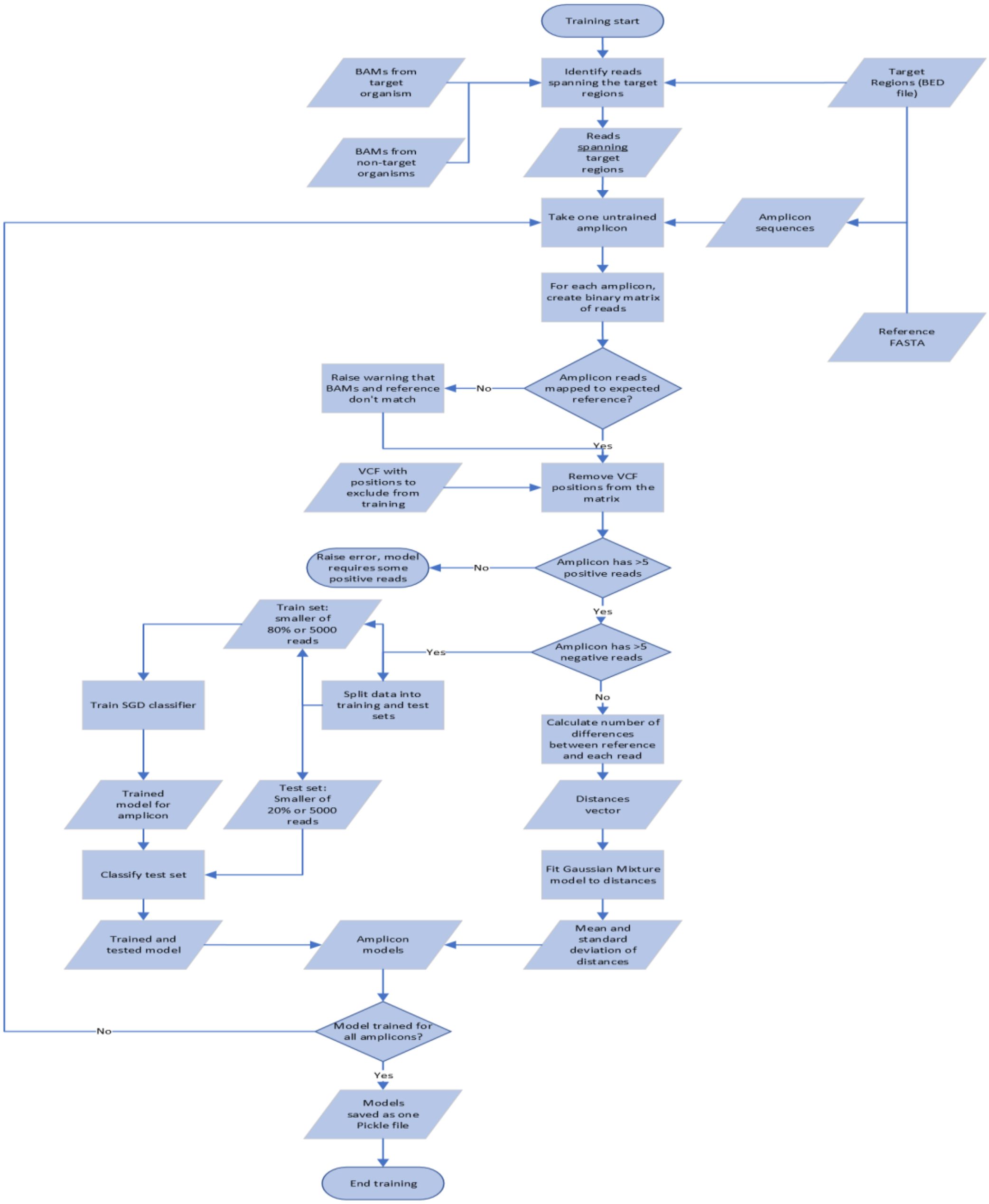

Supplementary Figure 1. Training workflow.

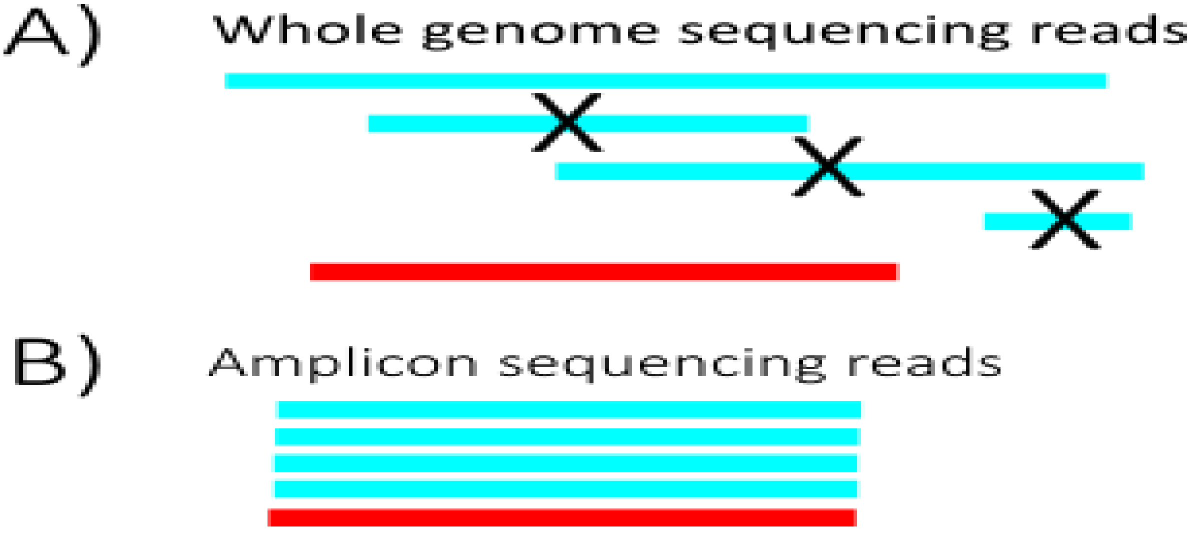

Supplementary Figure 2. Stylised distribution of reads (blue) against target amplicon (red) in whole genome and amplicon sequencing. Only reads that fully span the target amplicon are used for model training.

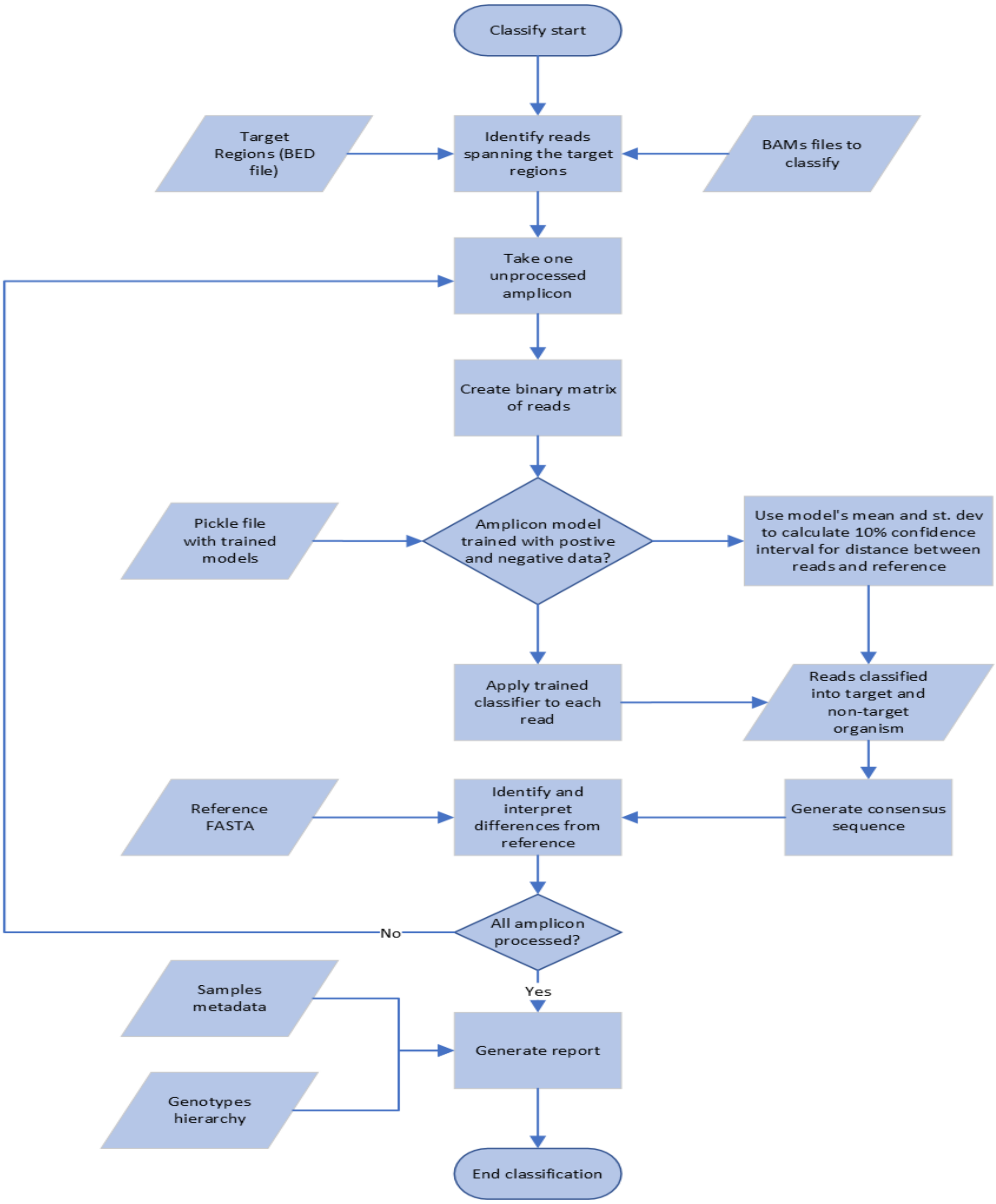

Supplementary Figure 3. Classification work flow
